# *β*-Aminoisobutyric Acid Relates to Favorable Glucose Metabolism through Adiponectin in Adults with Obesity Independent of Prediabetes

**DOI:** 10.1155/2023/4618215

**Published:** 2023-09-21

**Authors:** Habiba Faiz, Emily M. Heiston, Steven K. Malin

**Affiliations:** ^1^Rutgers University, New Brunswick, NJ, USA; ^2^University of Virginia, Charlottesville, VA, USA; ^3^Virginia Commonwealth University, Richmond, VA, USA; ^4^Division of Endocrinology, Metabolism & Nutrition, Rutgers University, New Brunswick, NJ, USA; ^5^New Jersey Institute for Food, Nutrition and Health, Rutgers University, New Brunswick, NJ, USA; ^6^Institute of Translational Medicine and Science, Rutgers University, New Brunswick, NJ, USA

## Abstract

*β*-Aminoisobutyric acid (BAIBA) is secreted by skeletal muscle and promotes insulin sensitivity, fat oxidation, and anti-inflammation. While BAIBA is purportedly lower in individuals with obesity, no work has examined if prediabetes (PD) differentially impacts BAIBA concentrations in people with obesity. *Methods*. Adults were classified as normal glucose tolerant (NGT; *n* = 22 (20F); 48.0 ± 2.4 yrs; 36.9 ± 1.2 kg/m^2^) or PD (*n* = 23 (18F); 54.2 ± 1.6 yrs; 38.4 ± 1.2 kg/m^2^) based on ADA criteria. A 180-minute 75 g OGTT was used to estimate fasting (HOMA-IR (liver)) and postprandial (Matsuda index (muscle)) insulin sensitivity as well as *β*-cell function (disposition index (DI), glucose-stimulated insulin secretion adjusted for insulin sensitivity). Body composition and fasting measures of BAIBA, fat oxidation (indirect calorimetry), and adipokines were determined. *Results*. NGT and PD had similar BAIBA concentrations (1.4 ± 0.1 vs. 1.2 ± 0.1 *μ*M, *P* = 0.23) and fat oxidation (*P* = 0.31), despite NGT having lower fasting (92.2 ± 1.2 vs. 104.1 ± 3.2 mg/dL, *P* = 0.002) and tAUC_180min_ glucose (*P* < 0.001) compared to PD. Moreover, NGT had higher postprandial insulin sensitivity (*P* = 0.01) and higher total phase DI_liver_ (*P* = 0.003) and DI_muscle_ (*P* = 0.001). Increased BAIBA was associated with adiponectin (*r* = 0.37, *P* = 0.02), adiponectin/leptin ratio (*r* = 0.39, *P* = 0.01), and lower glucose and insulin at 180 minutes (*r* = −0.31, *P* = 0.03 and *r* = −0.39, *P* = 0.03, respectively). Adiponectin also correlated with lower glucose at 180 minutes (*r* = −0.45, *P* = 0.005), and mediation analysis showed that BAIBA was no longer a significant predictor of glucose at 180 minutes after controlling for adiponectin (*P* = 0.08). *Conclusion*. While BAIBA did not differ between NGT and PD, higher BAIBA is related to favorable glucose metabolism, possibly through an adiponectin-related mechanism. Additional work is required to understand how exercise and/or diet impact BAIBA in relation to type 2 diabetes risk.

## 1. Introduction

Obesity is a public health problem in the United States [1] because it is related to higher mortality rates [2]. According to the CDC, obesity prevalence in the U.S is about 42% (BMI ≥ 30 kg/m^2^) among adults (NHANES 2021) [3, 4]. Two common characteristics of obesity are overnutrition and low physical activity levels [5]. These factors are important in influencing adipocyte function, namely, fat storage, substrate availability for energy metabolism, and endocrine function [6]. This later point is germane as adipose tissue secretes hormones known as adipokines, such as adiponectin and leptin, that have been used as indices of “sick fat” or adiposopathy [7]. Adiponectin is an anti-inflammatory adipokine that is low in circulation among adults with obesity. This is relevant because adiponectin promotes insulin sensitivity, *β*-cell function, and fat oxidation, in part, by activating AMPK [8]. In contrast, leptin regulates satiety and energy expenditure and is related to fat mass [9]. Thus, understanding how adiposopathy may be influenced could play an important role in chronic disease risk since adipose tissue has crosstalk with other organs including skeletal muscle [7] to influence insulin sensitivity [10].

Skeletal muscle is an important endocrine organ in the body that influences whole-body energy metabolism and insulin sensitivity [11]. Hormones secreted from the skeletal muscle are called myokines, and one such myokine that has gained recent attention is *β*-aminoisobutyric acid (BAIBA). BAIBA is a nonprotein amino acid released from skeletal muscle in response to contraction through the upregulation of PGC-1*α* [12]. Low BAIBA concentrations have been linked to elevated cardiometabolic risk factors in humans, namely, blood glucose [12]. Interestingly, mice treated with BAIBA show reductions in weight and body fat [12]. Further, BAIBA-treated human pluripotent stem cells demonstrate weight reduction in relation to enhanced fat oxidation, aerobic fitness, and insulin sensitivity, as well as reduced hepatic de novo lipogenesis [12]. This is consistent with other work in rats treated with BAIBA showing improved glucose tolerance, lower circulating lipid levels, and attenuated diet-induced obesity [13]. While the exact mechanism is unclear, BAIBA may exert these beneficial effects either directly on target organs or through adiponectin-/leptin-dependent pathways [13]. To date, however, no studies exist in humans that have tested if circulating BAIBA levels are different in people with obesity who are normal glucose tolerant (NGT) compared with those with prediabetes (PD) in relation to adiposopathy, insulin sensitivity, or energy metabolism. Therefore, we tested the hypothesis that BAIBA would be lower in people with PD compared to those with NGT. We anticipated that lower BAIBA levels would correlate with reduced insulin sensitivity, fat oxidation, and glucose tolerance.

## 2. Methods

### 2.1. Participants

A cross-sectional study was conducted in people with overweight/obesity who either were NGT (*n* = 22 (20F/2M), 48.0 ± 2.4 yrs, 36.8 ± 1.1 kg/m^2^) or PD (*n* = 23 (18F/5M), 54.2 ± 1.6 yrs, 38.4 ± 1.2 kg/m^2^) (Table 1). Glucose tolerance classification was based on American Diabetes Association (ADA) criteria and included those with impaired fasting glucose (IFG, 100-125 mg/dL), impaired glucose tolerance (IGT, 140-199 mg/dL), and/or IFG+IGT. Participants were eligible if ≥18 yrs of age, had a body mass index (BMI) of 25-50 kg/m^2^, nonsmoking, physically inactive (≤60 minutes of exercise/week), had no history of chronic diseases (i.e., renal, hepatic, cardiovascular, and type 2 diabetes (T2D)), not taking medications that influence glycemia/insulin sensitivity (e.g., metformin and SGLT-2 inhibitors), and weight stable over the past 3 months (≤2 kg variation). Participants underwent a medical examination conducted by a study physician as well as resting and maximal stress test electrocardiogram with blood pressure to assess cardiac function. Additionally, urine and blood samples were collected for biochemical analysis. Lastly, blood pressure was recorded 3 times with 1–2-minute rest in between (systolic ≥ 130 mmHg and/or diastolic ≥ 85 mmHg) in a seated position with feet flat on the floor and averaged. Written and verbal informed consent was obtained from all individuals before participating in the study. Study protocols conformed to the standards set by the latest version of the Declaration of Helsinki except for registration in a database and were approved by the University Institutional Review Boards (IRB #18316 and # Pro2020002029).

### 2.2. Aerobic Fitness

A continuous treadmill or bicycle incremental exercise test with indirect calorimetry was performed to assess respiratory gases for peak oxygen consumption (VO_2_peak) determination. Individuals self-selected a walking speed with incremental grade increasing approximately 2% every 2 minutes until exhaustion (NGT *n* = 8 vs. PD *n* = 16) or maintained a cadence above 60 rpm with increasing 25 W workload every 2 minutes (NGT *n* = 14 vs. PD *n* = 7). Heart rate (HR) and rating of perceived exertion (RPE) scale were also measured.

### 2.3. Body Composition

A digital scale was used to measure total weight while participants were without shoes and had minimal clothing. Height was measured using a stadiometer, and body mass index (BMI) was calculated as weight divided by height [2]. Waist circumference was obtained 3 times using a plastic tape measure 2 cm above the umbilicus and averaged. Body fat mass (FM) and fat-free mass (FFM) were determined by dual-energy X-ray absorptiometry (NGT *n* = 8 vs. PD *n* = 16; DXA; Horizon DXA System, Marlborough, MA, USA) or via air displacement plethysmography (NGT *n* = 14 vs. PD *n* = 7; BodPod, Concord, CA, USA) after a 4 hr fast (food and beverage) along with minimal physical activity for at least 4 hr [14, 15].

### 2.4. Metabolic Control

Participants were asked to refrain from nonexercise strenuous physical activity along with consumption of caffeine, alcohol, and medications 24 hr prior to the metabolic study visits. Participants were also advised to consume a mixed meal (e.g., 55% CHO, 30% fat, and 15% protein) the day prior to metabolic tests to standardize diet.

### 2.5. Insulin Action

Participants arrived at the Clinical Research Unit after an overnight fast to assess glucose metabolism and insulin action using a 180-minute75 g oral glucose tolerance test (OGTT). Indirect calorimetry (Vmax Encore, CareFusion, Yorba Linda, CA, USA) was used after approximately 20 minutes of rest in a semisupine position to assess resting energy expenditure (REE), respiratory exchange ratio (RER), and fat oxidation (F_ox_) and carbohydrate oxidation (CHO_ox_). Breath samples were collected for 15 minutes, and the last 5 minutes of data collected was averaged. Blood samples were then obtained at 0, 30, 60, 90, 120, and 180 minutes following a 75 g OGTT to determine glucose, insulin, and C-peptide. Total area under the curve (tAUC) was calculated using the trapezoidal rule. For peripheral, or skeletal muscle insulin sensitivity, the Matsuda index was calculated [16] while hepatic insulin resistance (HOMA-IR) was estimated by using fasting glucose multiplied by insulin divided by 405 [17]. Prehepatic glucose-stimulated insulin secretion (GSIS) was calculated as C-peptide tAUC divided by glucose tAUC during the OGTT to depict early (0-30 minutes) and total phase (0-180 minutes) insulin section. The disposition index was then used to calculate *β*-cell function relative to skeletal muscle as tAUC of GSIS^∗^Matsuda index*. β*-Cell function adjusted for hepatic insulin resistance was also calculated as GSIS divided by HOMR-IR. Hepatic insulin clearance (HIC) was calculated as tAUC of C-peptide divided by insulin during the OGTT [18].

### 2.6. Biochemical Analyses

Plasma glucose was analyzed immediately by a glucose oxidase assay (YSI Instruments 2700, Yellow Springs, OH). All remaining samples were centrifuged for 10 minutes at 1500 g at 4°C, and plasma was stored at -80°C until later batched-analyzed in duplicate. Insulin, C-peptide, total and HMW adiponectin, and leptin were measured from 3 mL EDTA vacutainers in-house using ELISA (EMD Millipore, Billerica, MA, USA) [19], while university medical laboratory performed colorimetric assay to analyze serum TG and cholesterol [20]. Adiponectin (total and HMW) and leptin were collected at 0 minute of the OGTT to depict adipocyte dysfunction, as we previously performed [19]. Specifically, adiposopathy was determined by dividing adiponectin by leptin. BAIBA was also collected in 3 mL EDTA vacutainers and measured by liquid chromatography mass spectrometry (LCMS) [21]. Briefly, 20 *μ*L of plasma sample was spiked with an internal standard solution consisting of isotopically labeled amino acid. The supernatant was immediately derivatized with 6-aminoquinolyl-N-hydroxysuccinimidyl carbamate according to Waters' MassTrak kit. A 10-point calibration standard curve underwent a similar derivatization procedure after the addition of internal standards. Both derivatized standards and the samples were analyzed on a Thermo Quantum Ultra triple quadrupole mass spectrometer coupled with a Waters Acquity liquid chromatography system. Data acquisition was done using select ion monitor (SIM) via positive electrospray condition. Concentration of the analyte was calculated against its respective calibration curve [21, 22].

### 2.7. Statistical Analyses

Data were analyzed using IBM SPSS Statistics (version 28.0.0.0 (190), IBM Corp). Normality was assessed and skewed data were log transformed. Logged data were assessed again to confirm normality. Unpaired two-tailed *t*-tests were used to compare group variables between NGT and PD. Log-transformed outcomes are presented in raw data format for ease of interpretation. Pearson or Spearman rank order correlation analyses were also performed to examine significant correlations between BAIBA, adipokines, and other variables when appropriate. Mediation regression analysis was used to investigate the hypothesis that fasting adiponectin concentrations mediate the effect of fasting BAIBA on glucose levels at 180 minutes. The indirect effect was tested using a percentile bootstrap estimation approach with 5000 samples and implemented with the PROCESS macro version 4 by Hayes. Mediation regression analysis was used to gain better insight on the relationship between BAIBA, adiponectin, and glucose at 180 min compared to a simple regression because of the correlation present between BAIBA and adiponectin with plasma glucose [23]. Significance was accepted as *P* ≤ 0.05, and data are presented as mean ± SEM.

## 3. Results

### 3.1. Participant Demographics

These data were presented as an abstract at the 45^th^ Annual Mid-Atlantic Regional Chapter of American College of Sports Medicine (MARC-ACSM) Conference in 2022 [24]. Although preliminary analysis with free fatty acids was reported at the MARC-ACSM conference, technical issues with kit availability did not allow for the entire sample size to be analyzed. This resulted in us not reporting free fatty acids here. Relevant to this work, free fatty acids did not relate to BAIBA. Nonetheless, there was no significant difference between NGT and PD with regard to weight, BMI, body composition, or aerobic fitness (Table 1). However, participants with PD were older (48 ± 2.3 vs. 54.2 ± 1.5, *P* = 0.03) compared to people with NGT (Table 1). Moreover, individuals with PD had higher HbA_1_c (*P* = 0.01) and TG (*P* = 0.02). HDL and LDL levels were not different between the groups, nor were fasting RER, CHO_ox_, or F_ox_ (Table 2).

### 3.2. BAIBA and Adipokines

**Figure 1:**
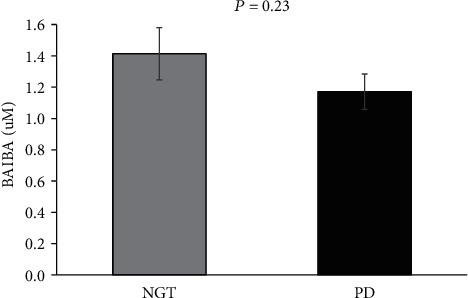
*β*-Aminoisobutyric acid (BAIBA) levels among NGT and PD. Data are presented as mean ± SEM. NGT = normal glucose tolerant; PD = prediabetes. Unpaired two-tailed *t-*test was utilized to determine group differences.

There was no significant difference in BAIBA concentrations between NGT and PD (Figure 1). Also, no significant differences were observed among leptin, total adiponectin, HMW adiponectin, or adiposopathy (Table 3).

### 3.3. Insulin Action

In line with higher fasting glucose (*P* = 0.002), people with PD had significantly higher glucose at 120 minutes (*P* = 0.01) and tAUC_180min_ glucose (*P* < 0.001) compared with NGT (Table 4). Fasting C-peptide (*P* = 0.001), tAUC_180min_ (*P* = 0.02), and insulin at 120 minutes (*P* = 0.02) were also significantly elevated in PD versus NGT (Table 4). Peripheral insulin sensitivity (*P* = 0.005) was higher in NGT compared with PD (Table 5). While early and total phase GSIS was not significantly different between the two groups, total phase DI_muscle_ (*P* = 0.003) and early as well as total phase DI_liver_ (both *P* = 0.001) were significantly higher in NGT as compared to PD (Table 5).

### 3.4. Correlations

**Figure 2:**
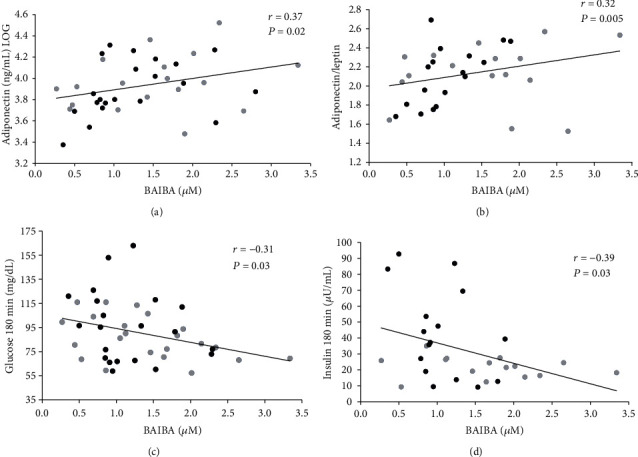
Correlations between BAIBA and (a) adiponectin, (b) adiposopathy, (c) glucose at 180 minutes, and (d) insulin at 180 minutes. Significance accepted at *P* ≤ 0.05. Grey circles, NGT; black circles, PD.

BAIBA did not correlate with F_ox_ (*r* = −0.002, *P* = 0.99) or skeletal muscle insulin sensitivity (*r* = 0.26, *P* = 0.11). However, higher BAIBA levels correlated with VO_2_peak (*r* = 0.33, *P* = 0.04), total adiponectin (*r* = 0.37, *P* = 0.02), HMW adiponectin (*r* = 0.322, *P* = 0.04), and total adiponectin/leptin ratio (*r* = 0.32, *P* = 0.05). Further, BAIBA was related to lower levels of glucose (*r* = −0.32, *P* = 0.03) and insulin (*r* = −0.39, *P* = 0.03) at 180 minutes each (Figures 2(a)–2(d)). Total adiponectin was inversely associated to glucose at 180 minutes (*r* = −0.45, *P* = 0.004) and glucose tAUC_180min_ (*r* = −0.40, *P* = 0.01), while both HMW and total adiponectin positively correlated to total phase DI_muscle_ (*r* = 0.435, *P* = 0.03 and *r* = 0.585, *P* = 0.002, respectively).

### 3.5. Mediation Analysis

**Figure 3:**
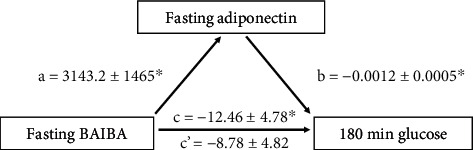
Simple mediation diagram. *a*, *b*, *c*, and *c*′ are path coefficients representing the unstandardized regression weights and standard errors. The *c* path coefficient represents the total effect of fasting BAIBA concentration on glucose levels at 180 minutes. The *c*-prime path coefficient refers to the direct effect of fasting BAIBA concentration on glucose at 180 minutes. ^∗^Significance accepted at *P* ≤ 0.05.

BAIBA was a significant predictor of adiponectin (*B* = 3143.2, SE = 1465.2, 95% CI [168.5, 6117.8], *β* = 0.34, *P* = 0.039), and adiponectin was a significant predictor of glucose levels at 180 minutes (*B* = −0.0012, SE = 0.0005, 95% CI [-0.0022, -0.0001], *β* = −0.35, *P* = 0.0319). Fasting BAIBA was no longer a significant predictor of glucose at 180 minutes after controlling for fasting adiponectin (*B* = −8.78, SE = 4.82, 95% CI [-18.57, 1.01], *β* = −0.28, *P* = 0.077) (Figure 3), consistent with a mediation effect. Approximately 27% of the variance of glucose at 180 minutes was accounted for by the predictors (*R*^2^ = 0.27), indicating a significant indirect coefficient (*B* = −3.68, SE = 2.42, 95% CI [-9.77, -0.25], completely standardized *β* = −0.12).

## 4. Discussion

The main finding of the present study is that PD status does not differentially impact BAIBA levels among people with obesity. However, BAIBA did relate to lower postprandial glucose concentrations as well as adiposopathy. In particular, adiponectin appeared to mediate the effect of BAIBA to impact postprandial glucose. This suggests that BAIBA may be a myokine that interacts with adipose tissue to establish crosstalk and impact whole-body glucose homeostasis. These findings are consistent with prior work highlighting lower BAIBA levels in adults with diabetes who were hemodialysis (HD) patients [25] as well as those people with obesity [26]. Why we did not detect lower BAIBA levels in people with PD compared with NGT is beyond the scope of the current study, but a potential reason is prior work included older adults or HD patients with diabetes and differing levels of BMI and comorbidities. Furthermore, these studies did not specifically control physical activity levels. Herein, all participants were sedentary and reasonably similar in key demographics, including adiposity and aerobic fitness, allowing for direct assessment of PD impact on BAIBA. Nonetheless, it is important to note that BAIBA exists in different isoforms. In particular, R-BAIBA dominate in both urine and plasma [27] and produced and degraded in the liver and the kidney, while S-BAIBA is produced and degraded primarily in the skeletal muscle, the brain, and the liver [28]. Thus, it is possible that subforms contribute differently, and additional work is required. Regardless, total BAIBA has been associated with reduced cardiometabolic risk [12] as well as plasma insulin levels [12]. This later point is consistent with our current work showing that higher BAIBA levels correlated with lower circulating glucose and insulin at 180 minutes of the OGTT in adults with obesity, independent of PD status. Thus, BAIBA appears to be a potential myokine that contributes to T2D risk.

BAIBA has been implicated as a potential target for lowering T2D risk [29]. In fact, BAIBA treatment in 6-week-old mice improved glucose tolerance during intraperitoneal glucose tolerance (IPGTT). Moreover, oral administration of BAIBA for 4 weeks in type 2 diabetic mice resulted in improved insulin signaling, by restoring Akt-IRS-1 (Tyr632 and Ser307) phosphorylation [30]. While we did not observe relations directly with BAIBA and insulin sensitivity, higher BAIBA did correlate with lower circulating insulin levels at 180 minutes of the OGTT. This is consistent with work suggesting that high BAIBA associates with low glucose tAUC during OGTT in humans [25]. Furthermore, others suggest that low BAIBA during hyperglycemic infusion conditions was paralleled with lower insulin secretory function regardless of presence or absence of hyperglycemia and T2D [31]. BAIBA has also been shown to improve glucose metabolism in mice of diet or streptozotocin-induced diabetic conditions [31].

Adiposopathy, or adipose dysfunction/“sick fat,” has been suggested to be causal in the development of T2D and cardiovascular disease [32]. We show that circulating BAIBA in NGT and PD adults with obesity correlates with adiposopathy. Specifically, BAIBA is related to adiponectin but not leptin, suggesting that BAIBA impacts the production of an insulin-sensitizing and anti-inflammatory mechanism that could influence glucose homeostasis. Indeed, we report that BAIBA and adiponectin correlated to lower glucose levels at 180 minutes. Human-induced pluripotent stem cells (IPSC) treated with BAIBA differentiated into white adipocytes with some of the beige genotype-specific genes like UCP-1 and enhanced mitochondrial biogenesis, and the functional effects of this outcome were observed by enhanced glucose uptake [12]. Mediation analysis in the current work suggests that controlling for adiponectin nullified the relationship of BAIBA and glucose, indicating that BAIBA may indirectly impact circulating glucose through an adiponectin-specific mechanism. These findings are somewhat consistent with evidence that BAIBA improved glucose homeostasis in mice through browning of the fat tissue by a PPAR-*α*-mediated pathway [33]. Further, muscle-derived BAIBA is purported to impact white adipocytes through JAK, PKA, and AMPK pathways [34] for adipose browning, which can promote insulin-stimulated glucose uptake in adipocytes [35]. In humans, BAT is known to raise the expression of UCP-1 in inner mitochondrial membrane and oxidize substrates like glucose to increase metabolic rate [36]. Despite these observations in rodents, we observed no relation of BAIBA with fat or carbohydrate oxidation, suggesting that BAIBA did not influence fuel metabolism and energy expenditure in this study. Adiponectin though itself plays an important role in insulin resistance [37] and pancreatic function [38]. Results obtained from our study demonstrate that adiponectin correlated with total phase GSIS scaled to indices of skeletal muscle insulin sensitivity (i.e., the disposition index). This suggests that adiponectin may modulate circulating glucose through the synthesis of insulin granules during the postprandial state compared with readily available release (i.e., early phase). Indeed, studies have shown that adiponectin may influence *β*-cell function by enhancing insulin granule exocytosis or insulin gene expression [39] as well as through direct action on *β*-cell adiponectin receptors [40]. Thus, our work suggests that adiponectin may modulate the need of the pancreas to secrete insulin for skeletal muscle glucose uptake [41], and further investigation into this potential muscle-adipose crosstalk is warranted [42].

This study has limitations that may influence our interpretation. The modest sample size prohibits comparisons of sex and race within NGT and PD groups, thereby limiting the generalization of these findings. Based on our current work, with a mean difference of 0.24 *μ*M and standard deviation of 0.65 for BAIBA between groups, it was suggested that 116 people per group would be needed at a power of 0.8 and significance set to 0.05 to detect statistical differences between NGT and PD. Further, people with PD were categorized by IFG, IGT, or IFG+IGT. While literature suggests that prediabetic phenotypes are depicted by varying levels and origins of insulin resistance [43], we did not observe differences in BAIBA concentrations among these subgroups (data not shown). However, additional work is needed to confirm. Adults with PD were statistically older (~5 yrs) than those with NGT, and it is possible that older age could influence our results. However, it is difficult to ascertain the physiologic relevance of such chronologic impact on BAIBA, and we observed no correlation between age and BAIBA despite insulin sensitivity, glucose tolerance, and body composition generally declining with age [44, 45]. We also used OGTT instead of a euglycemic-hyperinsulinemic clamp to measure insulin sensitivity. However, by assessing C-peptides, we were able to identify potential influences and relationships with pancreatic function compared with calculations of insulin sensitivity. Given that neither were related to BAIBA, it would seem reasonable to conclude that BAIBA was not a primary factor regulating insulin metabolism. Nevertheless, this study also has several strengths. We screened people for PD using standard clinical tests and provide detailed estimates of liver and skeletal muscle insulin sensitivity [46] as well as C-peptide-derived pancreatic function (via the disposition index) indexes to understand the physiologic relevance of BAIBA in glucose regulation [47]. Furthermore, participants consumed mixed meals and were instructed to omit vigorous physical activity and medications 24 hours prior to testing to enhance confidence determining if BAIBA differed between groups.

## 5. Conclusion

In conclusion, our study suggests that BAIBA is similar in adults with obesity characterized as NGT or PD. However, BAIBA concentrations may be related to favorable glucose metabolism via an adiponectin-related mechanism and highlights the importance of muscle-adipose crosstalk. Further work is required to examine if exercise and/or caloric restriction improves BAIBA levels in relation to lowering cardiometabolic disease risk.

## Figures and Tables

**Table 1:** Participant characteristics.

	NGT	PD	*P* value
Demographics			
*N* (F/M)	22 (20/2)	23 (18/5)	
Age (yrs)	48 ± 2.3	54.2 ± 1.5	0.03
Weight (kg)	105.6 ± 3.8	107.9 ± 4.6	0.69
BMI (kg/m^2^)	36.8 ± 1.1	38.4 ± 1.2	0.38
FFM (kg)	56.5 ± 2.2	57.0 ± 2.5	0.87
Body fat (%)	45.6 ± 1.3	47.0 ± 1.1	0.45
VO_2_peak (mL/kg/min)	20.75 ± 1.3	21.14 ± 0.9	0.82
Cardiometabolic disease risk			
WC (cm)	111.9 ± 2.7	116.8 ± 3.0	0.24
SBP (mmHg)	127.8 ± 2.6	136.6 ± 4.2	0.09
DBP (mmHg)	73.3 ± 2.4	78.5 ± 3.0	0.19
TG (mg/dL)	122.4 ± 16.3	162.3 ± 17.9	0.02
HDL-c (mg/dL)	46.2 ± 1.9	49.1 ± 2.8	0.77
LDL (mg/dL)	132.5 ± 9.5	133.6 ± 7.0	0.60
HbA_1_c (%)	5.3 ± 0.0	5.60 ± 0.07	0.01

Data are presented as mean ± SEM. NGT = normal glucose tolerant; PD = prediabetes; BMI = body mass index; VO_2_peak = aerobic capacity relative to mean body weight (kg); FFM = fat-free mass; WC = waist circumference; SBP = systolic blood pressure; DBP = diastolic blood pressure; TG = triglycerides; HDL-c = high-density lipoprotein; HbA_1_c = hemoglobin A_1_c. Unpaired two-tailed *t*-test was utilized to determine group differences. Significance accepted at *P* ≤ 0.05.

**Table 2:** Fuel use among NGT and PD.

	NGT	PD	*P* value
Energy expenditure			
REE (kcal/day)	1243 ± 69.7	1231.6 ± 88	0.92
REE (kcal/kg/day)	11.4 ± 0.5	12.0 ± 0.8	0.58
Fuel use			
CHO_ox_ (mg/kg/min)	0.94 ± 0.09	0.97 ± 0.13	0.79
F_ox_ (mg/kg/min)	0.49 ± 0.04	0.55 ± 0.05	0.35
RER (a.u.)	0.83 ± 0.009	0.81 ± 0.01	0.29

Data are presented as mean ± SEM. NGT = normal glucose tolerant; PD = prediabetes; REE = resting energy expenditure; CHO_ox_ = carbohydrate oxidation; F_ox_ = fat oxidation; RER = respiratory exchange ratio. Unpaired two-tailed *t*-test was utilized to determine group differences. Significance accepted at *P* ≤ 0.05.

**Table 3:** Adipokines between NGT and PD.

	NGT	PD	*P* value
Adipokines (ng/mL)			
Total adiponectin^	9655.7 ± 1259	9520.8 ± 1216	0.50
HMW adiponectin	4270.8 ± 923	3683.01 ± 585	0.84
Leptin	76.1 ± 9.7	62.8 ± 7.5	0.87
Total adiponectin/leptin^	167.6 ± 25.0	180.6 ± 32.2	0.92
HMW adiponectin/leptin	72.4 ± 14.8	80.9 ± 27.4	0.61

Data are presented as mean ± SEM. NGT = normal glucose tolerant; PD = prediabetes; HMW = high molecular weight. ^Data were log-transformed for statistical analysis but are presented as raw data for ease of interpretation. Unpaired two-tailed *t*-test was utilized to determine group differences. Significance accepted at *P* ≤ 0.05.

**Table 4:** Glucose metabolism between NGT and PD.

	NGT	PD	*P* value
Glucose (mg/dL)			
Fasting	92.2 ± 1.1	104.1 ± 3.2	0.002
120 min	113.0 ± 3.0	133.5 ± 7.0	0.01
tAUC_30min_	3400.8 ± 73.0	3841.6 ± 120	0.003
tAUC_180min_	20922.5 ± 545.9	25633.3 ± 1099.2	<0.001
Insulin (uU/mL)			
Fasting	15.83 ± 2.0	26.0 ± 4.2	0.09
120 min	79.83 ± 10.3	133.8 ± 19.8	0.02
tAUC_30min_	1597.4 ± 164.7	2217.7 ± 407.5	0.41
tAUC_180min_	15238.8 ± 2134.0	19087.2 ± 2959.2	0.22
C-peptides (ng/mL)			
Fasting	2.19 ± 0.1	3.60 ± 0.3	0.001
120 min	8.76 ± 0.65	12.47 ± 1.13	0.01
tAUC_30min_	145.3 ± 11.3	180.2 ± 14.0	0.03
tAUC_180min_	1372.6 ± 84.3	1790.6 ± 143.1	0.02

Data are presented as mean ± SEM. NGT = normal glucose tolerant; PD = prediabetes; tAUC = total area under the curve. Unpaired two-tailed *t*-test was utilized to determine group differences. Significance accepted at *P* ≤ 0.05.

**Table 5:** Insulin sensitivity and secretion between NGT and PD.

	NGT	PD	*P* value
Insulin sensitivity			
HOMA-IR	3.68 ± 0.4	6.0 ± 1.2	0.49
Matsuda index	2.75 ± 0.2	1.86 ± 0.2	0.005
Insulin secretion			
GSIS_30min_	0.04 ± 0.002	0.05 ± 0.003	0.17
GSIS_180min_	0.06 ± 0.003	0.07 ± 0.006	0.38
DI_muscle30min_^	0.12 ± 0.01	0.26 ± 0.10	0.22
DI_muscle180min_	0.20 ± 0.01	0.12 ± 0.01	0.003
DI_liver30min_^	0.82 ± 0.05	−2.12 ± 0.06	<0.001
DI_liver180min_	0.02 ± 0.002	0.06 ± 0.02	0.001
HIC tAUC_30min_^	0.10 ± 0.008	0.17 ± 0.05	1.00
HIC tAUC_180min_^	0.0004 ± 0.0	0.0006 ± 0.0002	0.78

Data are presented as mean ± SEM. ^Data were log-transformed for statistical analysis but are presented as raw data for ease of interpretation. NGT = normal glucose tolerant; PD = prediabetes; HOMA-IR = homeostatic model assessment for insulin resistance; GSIS = glucose-stimulated insulin secretion; DI = disposition index; HIC = hepatic insulin clearance. Unpaired two-tailed *t*-test was utilized to determine group differences. Significance accepted at *P* ≤ 0.05.

## Data Availability

Data will be provided by the corresponding author upon reasonable request.

## References

[1] Frühbeck G., Busetto L., Dicker D. (2019). The ABCD of obesity: an EASO position statement on a diagnostic term with clinical and scientific implications. *Obesity Facts*.

[2] Atakan M. M., Koşar Ş. N., Güzel Y., Tin H. T., Yan X. (2021). The role of exercise, diet, and cytokines in preventing obesity and improving adipose tissue. *Nutrients*.

[3] (2021). National Health and Nutrition Examination Survey 2017–March 2020 Prepandemic Data Files Development of Files and Prevalence Estimates for Selected Health Outcomes. https://stacks.cdc.gov/view/cdc/106273.

[4] Fryar C. D., Carroll M. D., Ogden C. L. (2012). *Prevalence of Overweight, Obesity, and Extreme Obesity among Adults: United States, Trends 1960–1962 through 2009–2010*.

[5] Gómez-Ambrosi J., Salvador J., Páramo J. A. (2002). Involvement of leptin in the association between percentage of body fat and cardiovascular risk factors. *Clinical Biochemistry*.

[6] Chouchani E. T., Kajimura S. (2019). Metabolic adaptation and maladaptation in adipose tissue. *Nature Metabolism*.

[7] Romacho T., Elsen M., Röhrborn D., Eckel J. (2014). Adipose tissue and its role in organ crosstalk. *Acta Physiologica*.

[8] Stefan N., Stumvoll M. (2002). Adiponectin-its role in metabolism and beyond. *Hormone and Metabolic Research*.

[9] Tong Y., Xu S., Huang L., Chen C. (2022). Obesity and insulin resistance: pathophysiology and treatment. *Drug Discovery Today*.

[10] Dietze D., Koenen M., Röhrig K., Horikoshi H., Hauner H., Jr E. (2002). Impairment of insulin signaling in human skeletal muscle cells by co-culture with human adipocytes. *Diabetes*.

[11] Rodríguez A., Becerril S., Ezquerro S., Mendez-Gimenez L., Frühbeck G. (2017). Crosstalk between adipokines and myokines in fat browning. *Acta Physiologica*.

[12] Roberts L. D., Boström P., O’Sullivan J. F. (2014). *β*-Aminoisobutyric acid induces browning of white fat and hepatic *β*-oxidation and is inversely correlated with cardiometabolic risk factors. *Cell Metabolism*.

[13] Begriche K., Massart J., Abbey-Toby A., Igoudjil A., Lettéron P., Fromenty B. (2008). *β*-Aminoisobutyric acid prevents diet-induced obesity in mice with partial leptin deficiency. *Obesity*.

[14] Malin S. K., Remchak M. M. E., Smith A. J., Ragland T. J., Heiston E. M., Cheema U. (2022). Early chronotype with metabolic syndrome favours resting and exercise fat oxidation in relation to insulin-stimulated non-oxidative glucose disposal. *Experimental Physiology*.

[15] Gilbertson N. M., Eichner N. Z., Heiston E. M. (2019). A low-calorie diet with or without interval exercise training improves adiposopathy in obese women. *Applied Physiology, Nutrition, and Metabolism*.

[16] Matsuda M., DeFronzo R. A. (1999). Insulin sensitivity indices obtained from oral glucose tolerance testing: comparison with the euglycemic insulin clamp. *Diabetes Care*.

[17] Matthews D. R., Hosker J. P., Rudenski A. S., Naylor B., Treacher D. F., Turner R. C. (1985). Homeostasis model assessment: insulin resistance and *β*-cell function from fasting plasma glucose and insulin concentrations in man. *Diabetologia*.

[18] Malin S. K., Rynders C. A., Weltman J. Y., Barrett E. J., Weltman A. (2016). Exercise intensity modulates glucose-stimulated insulin secretion when adjusted for adipose, liver and skeletal muscle insulin resistance. *PLoS One*.

[19] Heiston E. M., Eichner N. Z., Gilbertson N. M., Malin S. K. (2020). Exercise improves adiposopathy, insulin sensitivity and metabolic syndrome severity independent of intensity. *Experimental Physiology*.

[20] Malin S. K., Finnegan S., Fealy C. E., Filion J., Rocco M. B., Kirwan J. P. (2014). *β*-Cell dysfunction is associated with metabolic syndrome severity in adults. *Metabolic Syndrome and Related Disorders*.

[21] Wilkins J., Sakrikar D., Petterson X.-M., Lanza I. R., Trushina E. (2019). A comprehensive protocol for multiplatform metabolomics analysis in patient-derived skin fibroblasts. *Metabolomics*.

[22] Lanza I. R., Zhang S., Ward L. E., Karakelides H., Raftery D., Nair K. S. (2010). Quantitative metabolomics by 1H-NMR and LC-MS/MS confirms altered metabolic pathways in diabetes. *PLoS One*.

[23] Rijnhart J. J. M., Lamp S. J., Valente M. J., MacKinnon D. P., Twisk J. W. R., Heymans M. W. (2021). Mediation analysis methods used in observational research: a scoping review and recommendations. *BMC Medical Research Methodology*.

[24] Faiz H., Heiston E. M., Malin S. K. (2023). Beta-aminoisobutyric acid relates to favorable glucose metabolism and adiponectin in adults with metabolic syndrome. *International Journal of Exercise Sciences: Conference Proceedings*.

[25] Molfino A., Amabile M. I., Ammann T. (2017). The metabolite beta-aminoisobutyric acid and physical inactivity among hemodialysis patients. *Nutrition*.

[26] Short K. R., Chadwick J. Q., Teague A. M. (2019). Effect of obesity and exercise training on plasma amino acids and amino metabolites in American Indian adolescents. *The Journal of Clinical Endocrinology & Metabolism*.

[27] Stautemas J., Van Kuilenburg A. B., Stroomer L. (2019). Acute aerobic exercise leads to increased plasma levels of R-and S-*β*-aminoisobutyric acid in humans. *Frontiers in Physiology*.

[28] Tanianskii D. A., Jarzebska N., Birkenfeld A. L., O’Sullivan J. F., Rodionov R. N. (2019). Beta-aminoisobutyric acid as a novel regulator of carbohydrate and lipid metabolism. *Nutrients*.

[29] Guo A., Li K., Xiao Q. (2020). Sarcopenic obesity: myokines as potential diagnostic biomarkers and therapeutic targets?. *Experimental Gerontology*.

[30] Shi C.-X., Zhao M.-X., Shu X.-D. (2016). *β*-Aminoisobutyric acid attenuates hepatic endoplasmic reticulum stress and glucose/lipid metabolic disturbance in mice with type 2 diabetes. *Scientific Reports*.

[31] Barlow J. P., Karstoft K., Vigelsø A. (2020). Beta-aminoisobutyric acid is released by contracting human skeletal muscle and lowers insulin release from INS-1 832/3 cells by mediating mitochondrial energy metabolism. *Metabolism Open*.

[32] Hajer G. R., van Haeften T. W., Visseren F. L. J. (2008). Adipose tissue dysfunction in obesity, diabetes, and vascular diseases. *European Heart Journal*.

[33] Severinsen M. C. K., Schéele C., Pedersen B. K. (2020). Exercise and browning of white adipose tissue - a translational perspective. *Current Opinion in Pharmacology*.

[34] Chen W., Wang L., You W., Shan T. (2021). Myokines mediate the cross talk between skeletal muscle and other organs. *Journal of Cellular Physiology*.

[35] Stanford K. I., Middelbeek R. J., Townsend K. L. (2013). Brown adipose tissue regulates glucose homeostasis and insulin sensitivity. *The Journal of Clinical Investigation*.

[36] Maliszewska K., Kretowski A. (2021). Brown adipose tissue and its role in insulin and glucose homeostasis. *International Journal of Molecular Sciences*.

[37] Kadowaki T., Yamauchi T., Kubota N., Hara K., Ueki K., Tobe K. (2006). Adiponectin and adiponectin receptors in insulin resistance, diabetes, and the metabolic syndrome. *The Journal of Clinical Investigation*.

[38] Dunmore S. J., Brown J. (2013). The role of adipokines in b-cell failure of type 2 diabetes. *The Journal of Endocrinology*.

[39] Lee Y.-h., Magkos F., Mantzoros C. S., Kang E. S. (2011). Effects of leptin and adiponectin on pancreatic *β*-cell function. *Metabolism*.

[40] Kharroubi I., Rasschaert J., Eizirik D. L., Cnop M. (2003). Expression of adiponectin receptors in pancreatic *β* cells. *Biochemical and Biophysical Research Communications*.

[41] Berg A. H., Combs T. P., Du X., Brownlee M., Scherer P. E. (2001). The adipocyte-secreted protein Acrp30 enhances hepatic insulin action. *Nature Medicine*.

[42] de Oliveira dos Santos A. R., de Oliveira Zanuso B., Miola V. F. B. (2021). Adipokines, myokines, and hepatokines: crosstalk and metabolic repercussions. *International Journal of Molecular Sciences*.

[43] Malin S. K., Liu Z., Barrett E. J., Weltman A. (2016). Exercise resistance across the prediabetes phenotypes: impact on insulin sensitivity and substrate metabolism. *Reviews in Endocrine and Metabolic Disorders*.

[44] Kitase Y., Vallejo J. A., Gutheil W. (2018). *β*-Aminoisobutyric acid, l-BAIBA, is a muscle-derived osteocyte survival factor. *Cell Reports*.

[45] Guo X., Asthana P., Gurung S. (2022). Regulation of age-associated insulin resistance by MT1-MMP-mediated cleavage of insulin receptor. *Nature Communications*.

[46] Bastard J., Vandernotte J., Faraj M. (2007). Relationship between the hyperinsulinemic-euglycaemic clamp and a new simple index assessing insulin sensitivity in overweight and obese postmenopausal women. *Diabetes & Metabolism*.

[47] Faerch K., Brøns C., Alibegovic A. C., Vaag A. (2010). The disposition index: adjustment for peripheral vs. hepatic insulin sensitivity?. *The Journal of Physiology*.

